# Tackling the Biological Meaning of the Human Olfactory Bulb Dyshomeostatic Proteome across Neurological Disorders: An Integrative Bioinformatic Approach

**DOI:** 10.3390/ijms222111340

**Published:** 2021-10-20

**Authors:** Paz Cartas-Cejudo, Mercedes Lachén-Montes, Joaquín Fernández-Irigoyen, Enrique Santamaría

**Affiliations:** Clinical Neuroproteomics Unit, Navarrabiomed, Complejo Hospitalario de Navarra (CHN), Navarra Institute for Health Research, Universidad Pública de Navarra (UPNA), IdiSNA, 3, 31008 Pamplona, Spain; paz.cartas.cejudo@navarra.es (P.C.-C.); mercedes.lachen.montes@navarra.es (M.L.-M.); jfernani@navarra.es (J.F.-I.)

**Keywords:** olfactory bulb, neurodegeneration, proteomics, pathways

## Abstract

Olfactory dysfunction is considered an early prodromal marker of many neurodegenerative diseases. Neuropathological changes and aberrant protein aggregates occur in the olfactory bulb (OB), triggering a tangled cascade of molecular events that is not completely understood across neurological disorders. This study aims to analyze commonalities and differences in the olfactory protein homeostasis across neurological backgrounds with different spectrums of smell dysfunction. For that, an integrative analysis was performed using OB proteomics datasets derived from subjects with Alzheimer’s disease (AD), Parkinson’s disease (PD), mixed dementia (mixD), dementia with Lewy bodies (DLB), frontotemporal lobar degeneration (FTLD-TDP43), progressive supranuclear palsy (PSP) and amyotrophic lateral sclerosis (ALS) with respect to OB proteome data from neurologically intact controls. A total of 80% of the differential expressed protein products were potentially disease-specific whereas the remaining 20% were commonly altered across two, three or four neurological phenotypes. A multi-level bioinformatic characterization revealed a subset of potential disease-specific transcription factors responsible for the downstream effects detected at the proteome level as well as specific densely connected protein complexes targeted by several neurological phenotypes. Interestingly, common or unique pathways and biofunctions were also identified, providing novel mechanistic clues about each neurological disease at olfactory level. The analysis of olfactory epithelium, olfactory tract and primary olfactory cortical proteotypes in a multi-disease format will functionally complement the OB dyshomeostasis, increasing our knowledge about the neurodegenerative process across the olfactory axis.

## 1. Introduction

Neurodegenerative diseases are commonly characterized by a progressive loss of the structure and function of the central nervous system, mainly caused by a gradual neuronal loss. Among the more recurrent symptoms, this deterioration progressively causes a loss of cognitive abilities such as memory or decision-making, or movement disorders [1,2]. However, sleep disorders, constipation and olfactory dysfunction are increasingly being taken into account due to their great impact on the quality of life [3,4,5]. In fact, olfactory impairment is considered a prodromal sign of neurodegeneration and consequently, a reliable marker [5]. There is a spectrum of olfactory dysfunction ranging from severe loss, as observed in Alzheimer’s disease (AD) and Parkinson’s disease (PD), to little olfactory deficits (e.g., frontotemporal dementias (FTD)). In this sense, it has been suggested that there exists a common pathological substrate acting at the level of the olfactory system [6]. 

The olfactory bulb (OB) is the first site for the processing of olfactory information in the brain. Axons from olfactory receptor neurons exit the olfactory epithelium (OE), grow toward the brain, and penetrate the OB [7], where they synapse on the dendrites of mitral and tufted cells. The axons of these neurons then emerge from the OB, forming a discrete fiber bundle, the so-called olfactory tract (OT) [8]. These OT axons have collateral branches to the olfactory cortex where olfactory information is processed [9]. Hyposmia and anosmia result from changes at both the anatomical and the molecular level. OB atrophy as well as the reduction of cholinergic centrifugal inputs to the OB and the increased number of dopaminergic cells observed in this olfactory area have been suggested as potential origins of smell loss [10,11,12]. Interestingly, OTs undergo early and sequential morphological alterations that correlate with the development of dementia [13]. On the other hand, the characterization of the protein aggregates present in the OB and OT has revealed that the presence and severity of hyperphosphorylated tau, Aβ and α-synuclein pathology in both olfactory sites reflects the presence and severity of respective pathologies in other brain regions [14]. However, the comprehensive molecular profiling of the human OB during the progression of human NDs has received little attention. In this context, an in-depth biochemical characterization of the pathological neurodegeneration that occurs at the level of the OB has been performed by our group, partially revealing missing links in the biochemical understanding of the degeneration that accompanies the early smell impairment in distinct neurological disorders [15,16,17,18,19,20]. Due to the immense complexity that the study of the human brain entails, neuroproteomics has emerged as a powerful tool to profile neural/olfactory proteomes using shotgun-based mass spectrometry [21,22]. More importantly, the number of papers implementing proteomic approaches to understand the molecular background of neurological disorders has increased over the years [23,24], aiming to understand the molecular knowledge concerning the progression of neurological disorders. 

In view of the fact that the olfactory molecular analysis might help finding neuroprotective or even disease-modifying treatment strategies, this study aims to analyze commonalities and differences in the olfactory protein homeostasis across neurological backgrounds with different spectrums of smell dysfunction. For that, an integrative analysis was performed using OB proteomics datasets derived from subjects with Alzheimer´s disease (AD), Parkinson´s disease (PD), mixed dementia (mixD), dementia with Lewy bodies (DLB), frontotemporal lobar degeneration (FTLD-TDP43), progressive supranuclear palsy (PSP) and amyotrophic lateral sclerosis (ALS) with respect to OB proteome data from neurologically intact controls.

## 2. Results

Olfactory impairment is a common event during aging, and is aggravated in neurological disorders [6]. However, it has been observed that the olfactory loss is dependent on each neurodegenerative disease, ranging from severe (AD, PD, DLB, and mixD) to moderate (frontotemporal dementia) and mild (ALS and PSP). The OB is the first olfactory brain area where neuropathological changes and molecular alterations occur [14,25,26], being considered a site for prion-like propagation of pathological misfolded protein aggregates in neurological disorders [27,28]. In this descriptive study, OB proteome datasets were conjointly analyzed to deeply characterize potential common or unique molecular events across seven neurodegenerative diseases (AD, PD, DLB, MixD, FTLD-TDP43, ALS and PSP) that were previously analyzed in an independent manner [15,16,17,18,19,20]. For that, multi-level bioinformatics was applied to decipher proteostatic commonalities and specificities through a meta-analysis focused on pathway and biofunction mapping, protein–protein interactions and transcription factor prediction. 

### 2.1. Global Vision of the Proteostatic Derangements That Occur in the Olfactory Bulb across Neurodegenerative Diseases 

**Figure 1:**
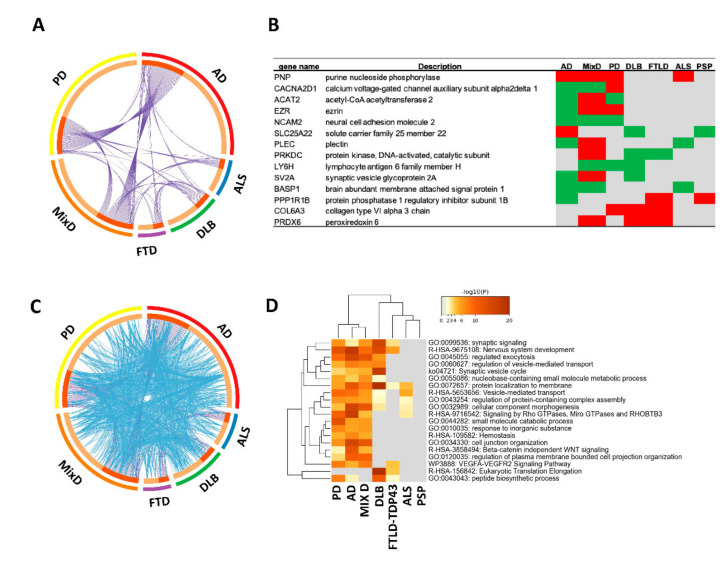
Overlap between olfactory bulb DEPs across neurodegenerative diseases. (**A**) Circos plot showing the OB proteome shared between neurological disorders. On the outside, each colored arc represents the identity and the dimension of each proteomic dataset. On the inside, dark orange color represents the proteins that appear in multiple datasets and light orange color represents unique deregulated proteins specific to each disease. Purple lines reflect the proteome that is shared across neurological phenotypes. (**B**) Table representing the most deregulated OB proteins across datasets (minimum in three neurological phenotypes). Green, red and gray colors indicate down-regulation, up-regulation and no change/no detection, respectively, in each dataset. (**C**) Circos plot in which blue lines link the DEPs where they fall into the same statistically significant ontology term. (**D**) Functional clustering of the top 20 pathways and biofunctions significantly enriched. The heatmap cells are colored by their *p*-values; gray cells indicate the lack of enrichment for that term in the corresponding protein dataset. See Appendix A for Metascape analysis details.

Taking into account all OB proteomic datasets, 812 differential expressed proteins (DEPs) with respect to non-demented controls were considered (FDR < 1%, *p* < 0.05 and fold-change: 30%). A total of 80% of the DEPs (659 proteins) were assigned to a specific disease, whereas the remaining 20% (153 proteins) corresponded to the OB dysregulated proteome detected in at least two neurodegenerative phenotypes (Figure 1A and Table S1). Purine nucleoside phosphorylase (*PNP*) was the unique overexpressed protein in four neurodegenerative diseases (AD, PD, MixD and ALS) (Figure 1B). Involved in the purine metabolism, this protein catalyzes the phosphorolysis of inosine, guanosine and their deoxynucleosides (https://www.uniprot.org/uniprot/P00491 accesed on 12 Semptember 2021), and its deficiency induces cerebellar abnormalities and progressive motor deficits in mice [29]. Moreover, deregulated *PNP* mRNA levels in specific brain areas have been previously associated with AD, PD and DLB [30,31,32], suggesting that purine metabolic enzymes are part of the global molecular machinery disrupted during neurodegeneration.

**Figure 2:**
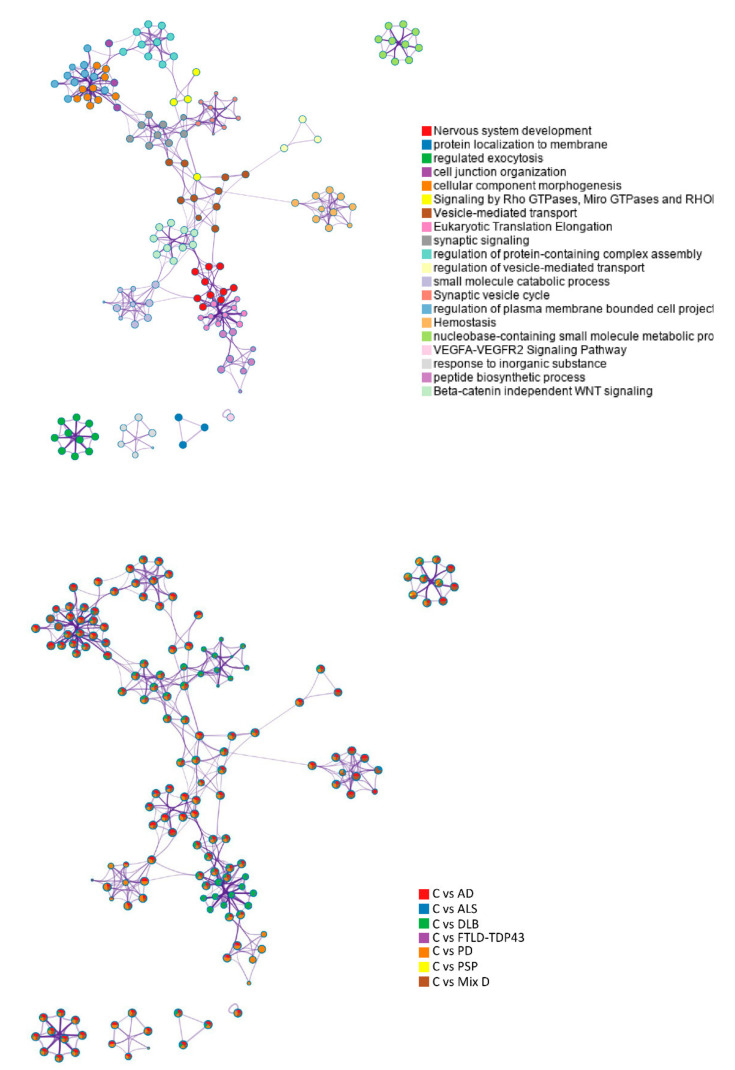
Enriched ontology clusters and functional connections. Groups of representative GO were converted into a network by Metascape [37]. Briefly, each GO term is represented by a circle node and its size is proportional to the number of DEPs that fall into each term. Nodes of the same color represent the same cluster. Thickness of the edge represents the similarity score (terms with a similarity score >0.3 are linked by an edge). Metascape selects one term from each cluster to label the term description. See Appendix A for Metascape analysis details.

Thirteen OB proteins were differentially regulated in at least three neurological disorders, of which four of them (*NCAM2*, *LY6H*, *COL6A3* and *PRDX6*) presented a homogeneous OB profile across diseases (Figure 1B). *NCAM2* (down-regulated in AD, MixD and PD) is a homophilic adhesion molecule expressed in sensory neurons with a potential role in specific fasciculation and zone-to-zone projection of the primary olfactory axons [33]. *LY6H* (down-regulated in MixD, PD and DLB) is a regulator of the alpha7 nicotinic acetylcholine receptor trafficking, a process involved in sensory processing [34]. *COL6A3* (up-regulated in PD, DLB and FTLD) is an extracellular matrix protein with neuronal protective roles under stress conditions [35]. It has been observed that *PRDX6* (up-regulated in MixD, DLB and FTLD) may play a dual role, attenuating the oxidative toxicity induced by pathological aggregates as well as regulating neuroinflammation, neurogenesis and the mitochondrial oxidative stress [36]. It is important to note that this low coincidence between DEPs may be due not only to the neurodegenerative process but also to the quantitative proteomic method used in each case (Table S1). However, a global functional overlap between OB dyshomeostatic proteome was clearly evidenced across neurodegenerative diseases (Figure 1C). Specifically, this significant functional overlap refers to bioprocesses such as synaptic signaling, exocytosis, protein localization to membrane, protein complex assembly, morphogenesis and VEGF signaling pathway (Figure 1D). As shown in Figure 2, most affected biofunctions are highly interconnected (Figure 2). All detailed information about functional annotations for each DEP is shown in Table S2.

**Figure 3:**
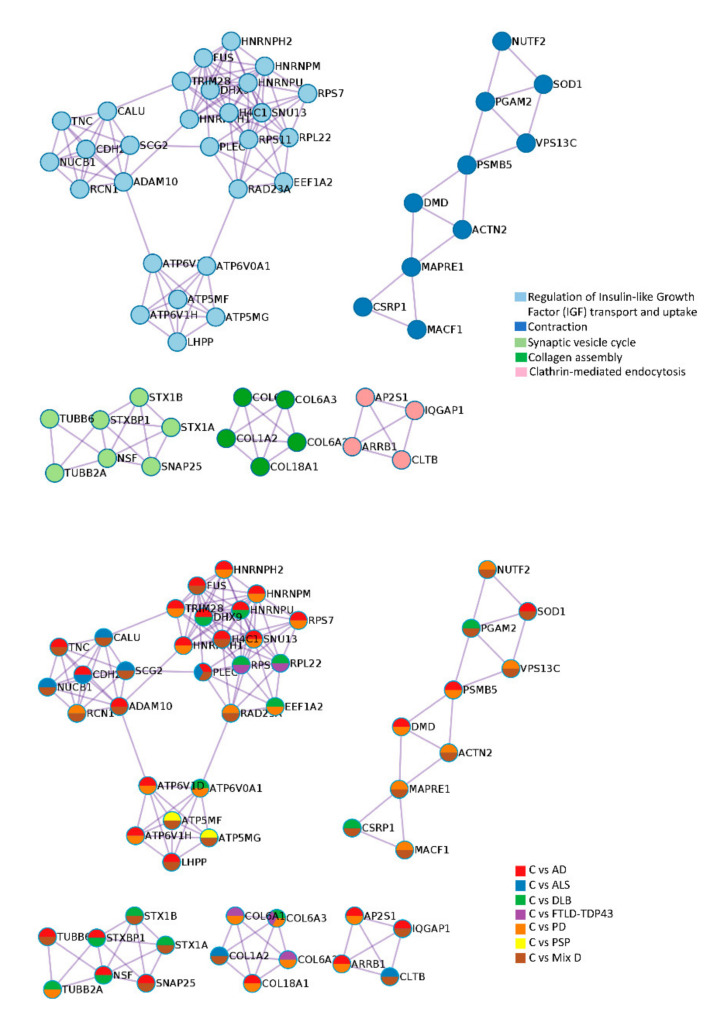
OB protein complexes dysregulated by several neurological disorders. Protein complexes embedded in proteomics outputs were automatically extracted using the MCODE algorithm [41]. Using Metascape, the three most significantly enriched ontology terms were combined to annotate putative biological roles for each MCODE complex (upper). Protein components of each complex differentially modulated across neurological disorders considered in our survey (lower). See Appendix A for Metascape analysis details.

In addition, our cross-disease analysis revealed that several protein complexes involved in IGF-1 regulation, contraction, synaptic vesicle cycle, collagen assembly and clathrin-mediated endocytosis were differentially targeted across neurodegenerative diseases (Figure 3). In particular, OB IGF-1 signaling was differentially affected by all diseases checked (Figure 3). This complex is highly relevant in axon guidance, the olfactory sensory map, neurogenesis and olfactory memory [38,39,40].

**Figure 4:**
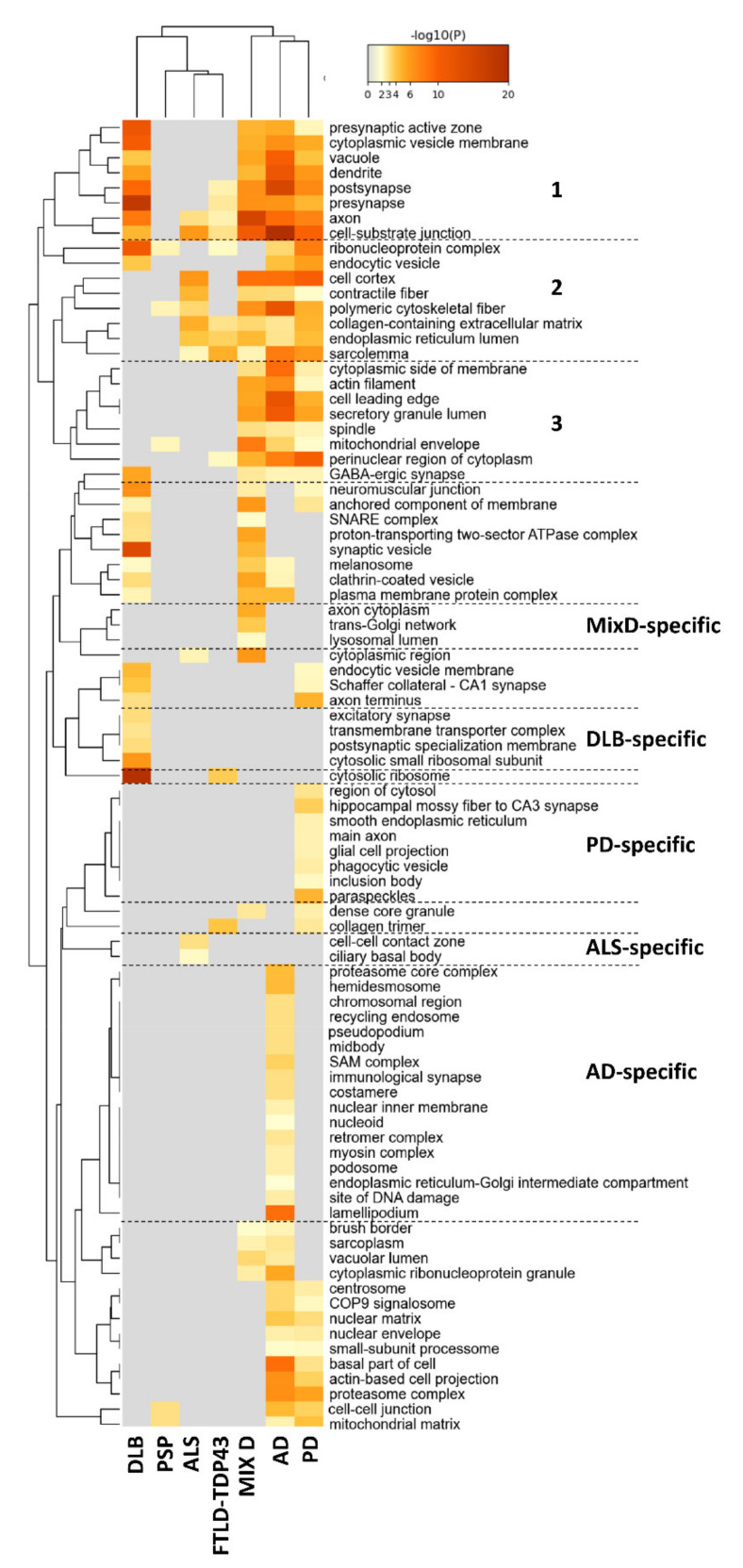
Subcellular mapping of OB DEPs across neurological disorders. Functional clustering representing cellular compartments significantly enriched. As mentioned above, heatmap cells are colored by their *p*-values; gray cells indicate the lack of enrichment for that term in the corresponding protein dataset. Cluster 1 corresponds to synaptic and axonal zones. Cluster 2 refers to ribonucleoprotein and cytoskeletal components. Secretory granules, spindles, mitochondrial envelope and GABAergic synapse were GO terms significantly affected in AD, PD and MixD (Cluster 3). See Appendix A for Metascape analysis details.

**Figure 5:**
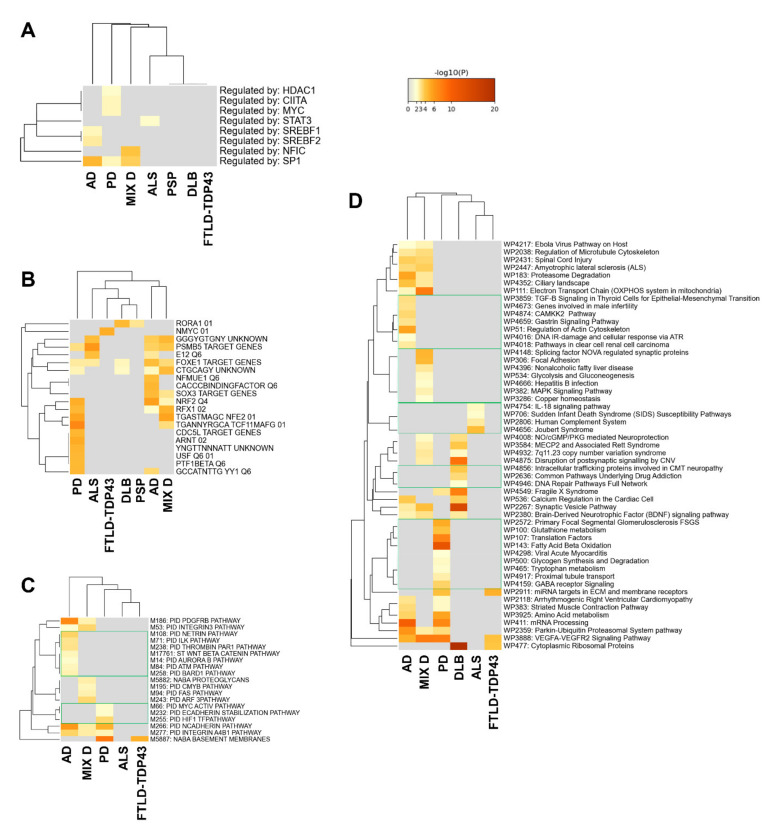
Potential transcription factors responsible for the OB proteostatic events detected across AD, PD, ALS, FTLD-TDP43, MixD, PSP and DLB. For that, TRRUST (**A**) and MsigDB algorithms (**B**) [42,43] integrated in the Metascape platform were used (Tables S3 and S4). Multi-disease pathway mapping using canonical pathways (**C**) and Wikipathways (**D**) (Tables S5 and S6). See Appendix A for Metascape analysis details.

**Figure 6:**
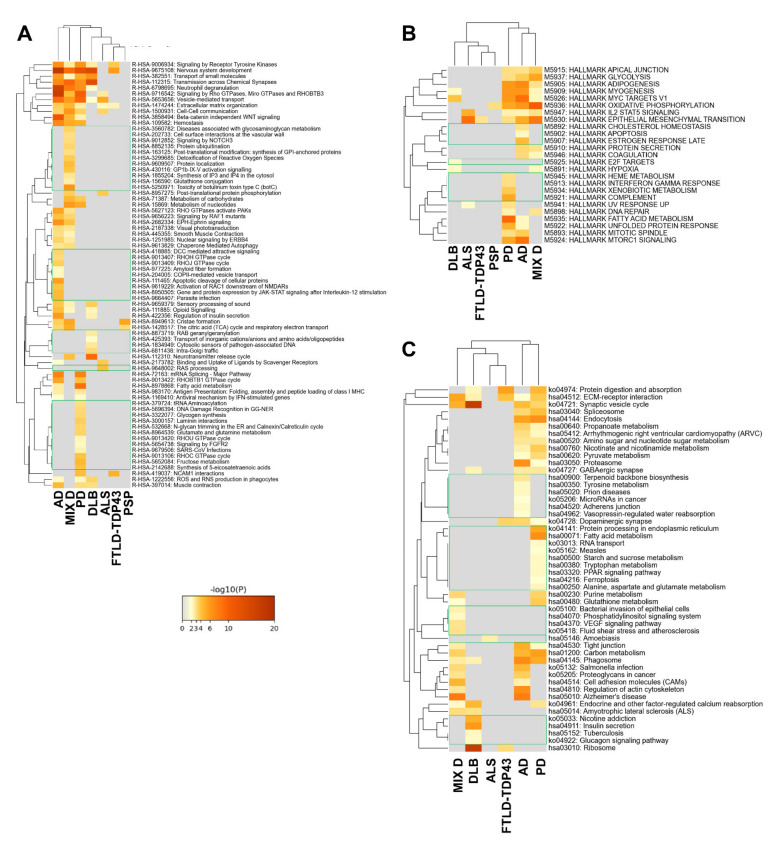
Multi-disease pathway mapping using Reactome (**A**), hallmark gene sets (**B**) and KEGG (**C**) (Tabels S7–S9).

We also explored the commonalities and differences at the level of organellar localization, transcription factors potentially responsible for the downstream effects detected at proteome level, and pathway enrichment clusters (Figure 4, Figure 5 and Figure 6).

Although multiple cellular locations presented a disease-specific significant alteration, the cellular areas most affected corresponded to synaptic and axonal zones (cluster 1 in Figure 4) together with ribonucleoprotein and cytoskeletal components (cluster 2 in Figure 4). Moreover, secretory granules, spindles, mitochondrial envelope and GABAergic synapse were GO terms significantly affected in AD, PD and MixD (cluster 3 in Figure 4). With respect to transcriptional regulation, multiple transcription factors could explain the OB proteomic changes. Specifically, SP1-regulated genes are significantly represented in AD, PD and MixD datasets (Figure 5A). According to MsigDB (Figure 5B), a subset of deregulated proteins observed in PD, ALS, AD and MixD present several binding sites for PSMB5 in their promoter regions. FOXE1 binding sites are also significantly over-represented in deregulated proteomes from PD, ALS, DLB, AD and MixD (Figure 5B). The most significant pathways represented in deregulated proteomes are: cadherin and integrin A4B1 pathways (Figure 5C), parkin-ubiquitin proteasomal route and VEGF signaling (Figure 5D), extracellular matrix regulation, vesicle-mediated transport, signaling by RHO GTPases, signaling by receptor tyrosine kinases and synaptic transmission (Figure 6A,C). As shown in Figure 6B, glycolysis and oxidative phosphorylation are also targeted by PD, AD and MixD.

### 2.2. Olfactory Bulb Proteomic Singularities Associated with Alpha Synucleinopathies: PD and DLB

PD induces specific proteostatic changes at the level of the main axon, glial cell projection, phagocytic vesicle, inclusion body and paraspeckles (Figure 4). At the transcriptional level, bioinformatics analysis predicted *HDAC1*, *CIITA*, *MYC*, *CDC5L*, *USF* and *PTF1* beta as potential regulators of the OB modulated proteome in PD cases (Figure 5A,B). Specifically, it has been observed that HDA1 inhibition potentiates cell death and CIITA is necessary for alpha-synuclein-induced MHC-II induction and subsequent peripheral immune cell infiltration in different PD models [44,45]. In line with our observations, part of the transcriptomic alterations in PD substantia nigra is controlled by CDC5L [46]. Using Canonical Pathways and Wikipathways, HIF1 signaling cascade, fatty beta oxidation, tryptophan metabolism and GABA receptor signaling were statistically significant overrepresented biofunctions in PD (Figure 5C,D). Moreover, other pathways such as glycogen synthesis, glutamate/glutamine and fructose metabolism, FGFR2 signaling, interferon gamma response, ER protein processing and PPAR signaling were exclusively enriched in PD and not in DLB, being a novel source of differential features across alpha synucleinopathies (Figure 6A–C). On the other hand, subcellular analysis revealed that DLB induces significant proteomic changes at the level of excitatory synapse, transmembrane transporter complexes and cytosolic small ribosomal subunits (Figure 4), converging in specific pathways such as neuropathic protein trafficking, Rag geranylgeranylation, transport of inorganic cations/anions, nicotine addiction and insulin secretion (Figure 5D and Figure 6A,C).

### 2.3. Olfactory Bulb Proteomic Singularities Associated with AD

As shown in Figure 4, multiple GO subcellular terms were significantly and specifically affected in AD OBs. Some of them were retromer complex involved in endosome protein sorting, SAM complex related to protein assembly in the external mitochondrial membrane and specialized cytoskeletal structures such as lamellipodium and podosomes. SREBF1 and NFMUE1 (involving genes with 3’UTR containing motif CGGCCATCT) were the transcription factors specifically proposed as transcriptional mediators relevant in AD olfactory neurodegeneration (Figure 5A,B). *SREBF1*, involved in the active transcription of genes involved in cholesterol biosynthesis and lipid homeostasis, is activated in the presence of superoxide production and high concentrations of Aβ [47]. The multi-pathway tool used in this study allowed us to map multiple disease-specific routes. As shown in Figure 5, OB protein components involved in pathways with upstream regulators such as netrin, *ILK* (Integrin Linked Kinase), thrombin, Aurora B, *ATM* (ATM Serine/Threonine Kinase) and *CAMKK2* were specifically enriched in AD (Figure 5C,D). Specific mediators of RHO GTPase cycle, amyloid fiber formation, RAC1 activation, apoptosis, cholesterol homeostasis, adherens junction and tyrosine metabolism were also proposed by Reactome, hallmark gene sets and KEGG as significantly enriched pathways in AD datasets (Figure 6A–C).

### 2.4. Olfactory Bulb Proteomic Singularities Associated with MixD

As shown in Figure 4, proteins resident in trans-Golgi network, axonal cytoplasm and lysosomal lumen were specifically mapped in MixD. In this case, NFIC (Nuclear Factor I C) was the only transcription factor potentially involved in OB proteomic imbalance in this neurological phenotype (Figure 5A). Enrichment analysis pointed out that proteoglycans, ADP-ribosylation factor 3 (ARF3) pathway, NOVA-regulated synaptic proteins, NOTHC3 signaling, synthesis of GPI-anchored proteins, glutathione conjugation and phosphatidylinositol signaling were part of the biofunctions selectively enhanced in MixD cases (Figure 5C,D and Figure 6A,C).

### 2.5. Olfactory Bulb Proteomic Singularities Associated with Neurological Disorders Where Olfactory Loss Occurs to a Lesser Extent 

In ALS, deregulated proteins were mapped in cell–cell contact zones and ciliary basal bodies (Figure 4). These proteins were potentially regulated by STAT3 (Figure 5A) and mainly involved in IL-18 signaling and complement systems (Figure 5D), RAS processing and amoebiasis (Figure 6A,C). With respect to FTLD-TDP43, a specific enrichment of proteins regulated by NMYC was detected (Figure 5A). 

Although this bioinformatic analysis has revealed commonalities and differences in the OB dyshomeostatic proteome across several alpha-synucleinopathies, tauopathies and tardopathies, potential limitations warrant discussion. It is important to note that the proteomic datasets considered in this study were generated using different proteomic workflows and mass-spectrometers, indicating that this exploratory analysis needs to be interpreted with caution because proteome coverage may differ across studies. Moreover, due to the limited samples size in each study, sex-based analysis was not considered in our functional approach. Since sex differences are clearly evidenced in olfaction and neurodegeneration [48,49], the future characterization of the contribution of sex to olfactory molecular routes underlying diverse neurological phenotypes could be relevant for the correct implementation of personalized neurology. Nowadays, proteomic approaches do not allow quantifying full proteomes specially in brain tissue. This means that relevant protein mediators such as receptors and transmembrane proteins are underrepresented in the studies considered in our survey. Specifically, the olfactory receptor family deserves special attention. Although its transcripts have been observed in human OB [50], this protein family is considered the largest portion of the “missing proteome” because it does not have high-stringency evidence at the mass-spectrometric level, due to multiple physicochemical and biological reasons [51,52]. Moreover, our analysis is limited to the protein abundance average among the multiple OB cell layers. The future implementation of laser-capture microdissection in combination with single-cell transcriptomics/proteomics approaches will dramatically increase the understanding of the specific role of olfactory cell-layers during the neurodegenerative process.

## 3. Materials and Methods

### 3.1. Literature Search and Data Mining

A review of published proteomics work focused on human OB was conducted to assemble a dataset consisting of differential expressed proteins between non-demented controls and different tauopathies, synucleinopathies and tardopathies such as Alzheimer’s disease (AD), Parkinson’s disease (PD), mixed dementia (mixD), dementia with Lewy bodies (DLB), frontotemporal lobar degeneration (FTLD-TDP43), progressive supranuclear palsy (PSP), amyotrophic lateral sclerosis (ALS) and neurologically intact controls [15,16,17,18,19,20]. Omic studies performed at the olfactory level in animal models of neurodegenerative disorders were not included in our survey. In total, 75 human OBs were previously subjected to proteome-wide analysis using mass-spectrometry. These proteomic datasets correspond to the following identifiers: PXD021630, PXD005319, PXD011446, PXD016069, PXD008036 and PXD025368 deposited in the ProteomeXchange/PRIDE repository.

### 3.2. Bioinformatics

The identification of specifically dysregulated regulatory/metabolic networks was analyzed using Metascape [37]. This tool allows the combination of gene annotation, membership search, interactome characterizations and functional enrichments, facilitating comparative analysis across multiple independent omic experiments. Specifically, we have used different resources integrated in Metascape. For transcription factor enrichment analysis, TRRUST and MsigDB were used [42,53]. These analyses were complemented with a pathway mapping using Canonical Pathways, Wikipathways [54], Reactome [55], hallmark gene sets [53] and KEGG [56]. Output lists derived from these tools are presented in Tables S3–S9. 

## Supplementary Materials

The following are available online at https://www.mdpi.com/article/10.3390/ijms222111340/s1. Table S1: Information datasets, Metascape input and common and unique OB deregulated proteins across NDs; Table S2: Functional analysis of OB differentially expressed proteins; Table S3: Analysis with TRRUST algorithm; Table S4: Analysis with MsigDB; Table S5: Canonical pathway analysis; Table S6: Analysis with Wikipathways; Table S7: Analysis with Reactome; Table S8: Analysis with hallmark gene sets; Table S9: Analysis with KEGG.

## Appendix A

The step-by-step process of the Metascape analysis was as follows:

1. To submit multiple lists, we checked the corresponding box and dropped the file containing all gene names corresponding to differential expressed OB proteins previously observed in neurological phenotypes. In our case, this was an .xls file (See Table S1).
2. Select the species. In our case, we selected *Homo Sapiens*. The background gene list for enrichment analysis was the default corresponding to the whole genome. It is important to note that Metascape utilizes the hypergeometric test and Benjamini–Hochberg *p*-value correction to identify ontology terms that contain a statistically greater number of genes/proteins in common with an input list than expected by chance.
3. The “express analysis” route was used to generate the outputs shown in Figure 1, Figure 2 and Figure 3. These types of analyses may be found in the “report page button” or in a .zip file. Specifically, Circos plots (overlaps represented in Figure 1A,C) are in the “overlap circus” folder. The heatmap (Figure 1D) corresponds to the heatmap that represents the top 20 GO terms (present in the “Enrichment heatmap” folder). All information associated with the enriched ontology clusters (Figure 2) is located in the “Enrichment GO” folder in the .zip file. Metascape also represents protein–protein interactions (PPI). The integrated PPI database includes STRING, BioGrid, OmniPath and InWeb_DB. The tool also applies MCODE (a mature complex identification algorithm) to automatically extract protein complexes embedded in large networks. Then, through its functional enrichment analysis capability, Metascape automatically assigns putative biological roles of each MCODE complex (see Figure 3). The information associated with PPIs is located in the “Enrichment PPI” folder of the .zip file.
4. The “custom analysis” route was used to focus the analysis on specific ontologies/functionalities (ontology catalogs):
  - As shown in Figure 4, custom analysis was used to specifically characterize the organellar distributions across neurodegenerative diseases.
  - As shown in Figure 5A,B, custom analysis was used to predict/identify potential transcription factors involved in deregulated proteomes. For that, Metascape integrates TTRUST and MsigDB algorithms (Transcription_factor_targets and TRRUST outputs in the “enrichment QC” folder)
  - As shown in Figure 5C,D and Figure 6A–C, custom analysis was used to perform functional enrichment analysis focused on pathways using default ontologies (canonical pathways, Wikipathways, Reactome, hallmark gene sets and KEGG).

In our case, default analysis parameters were used: enriched terms to include ≥ three candidates, *p*-value ≤ 0.01 and enrichment factor ≥1.5. Please visit the Metascape website (http://metascape.org/gp/index.html#/main/step1) to see over 40 data sources that are integrated in Metascape to perform identifier conversions, gene annotations, membership search and enrichment analysis.
